# Intestinal Calculi Lodged in Diaphragmatic Curvature of the Colon

**Published:** 1883-04

**Authors:** Frank V. Walton

**Affiliations:** Surgeon to Columbia Veterinary College Hospital; New York City C. V. C. H.


New York City.

---

### VIII.—INTESTINAL CALCULI LODGED IN DIAPHRAGMATIC CURVATURE OF THE COLON.

BY FRANK V. WALTON, D.V.S.,

*Surgeon to Columbia Veterinary College Hospital.*

Was called on March 3d to see a bay horse somewhat advanced in years. When reached, the animal was found to be in great pain.

The previous history was that he had not passed his feces during the last twenty-four hours and had refused all food.

A diagnosis of impaction was rendered. Anodines cathartics and anemas were given without any satisfactory result. Hot blankets were also applied for eight hours in succession, but proved unsucessful. The animal, however, lived for three days, during which time he suffered considerable, although morphia was freely administered.

A necropsy was held a few hours after death. The intestines were greatly distended by gas and contained considerable fluid. Upon opening the great gut, a large calculus, weighing 7½ pounds, was found in the diaphragmatic curvature of the colon. Strange to say, the horse had not been subject to colics, but had had an attack about one year ago.

There was no doubt in the mind of any person who saw the animal during life, but that it was a case of impaction; but just what the cause was continued in doubt until the autopsy.

One of the students who saw the case thought he got a point of localized dullness, and returned a diagnosis of intestinal calculus, but as the tympanitis and fluid became greater, even this little sign rapidly disappeared.

The stone, when removed, was not one of the smooth variety commonly met with, but was rough and nodulated, somewhat like the mulberry calculi, commonly found in the human bladder. This roughened exterior was an apparent shell to an internal and harder substance. While drying, this outer coating which, when moist, was more pasty in consistency, became dry and brittle, and easily cracked off from the central body.

Although representing the irregular contour of the mulberry caculus of the bladder, it was not of that variety, the comparison referring only to the gross external appearance.

As yet the calculus has not become sufficiently dry and hard to make a section of the same, but later on we will give a more complete description of the appearance of the section.

New York City C. V. C. H., March, 1883.

